# Brucella Seroprevalence and Associated Risk Factors in Occupationally Exposed Humans in Selected Districts of Southern Province, Zambia

**DOI:** 10.3389/fpubh.2021.745244

**Published:** 2021-11-17

**Authors:** Melai Mubanga, Ruth L. Mfune, John Kothowa, Ahmed S. Mohamud, Chitalu Chanda, John Mcgiven, Flavien N. Bumbangi, Bernard M. Hang'ombe, Jacques Godfroid, Martin Simuunza, John B. Muma

**Affiliations:** ^1^Department of Disease Control, School of Veterinary Medicine, University of Zambia, Lusaka, Zambia; ^2^Department of Environmental Health, School of Medicine and Health Sciences, Eden University, Lusaka, Zambia; ^3^Department of Public Health, Michael Chilufya Sata School of Medicine, Copperbelt University, Kitwe, Zambia; ^4^Department of Animal Health and Livestock Development, Blantyre Agriculture Development Division (BLADD), Mpemba, Malawi; ^5^Faculty of Veterinary Medicine, Red Sea University, Galkaio, Somalia; ^6^Infectious Diseases Unit, Department of Internal Medicine, The University Teaching Hospital, Lusaka, Zambia; ^7^Animal and Plant Health Agency Woodham Lane, New Haw Surrey, United Kingdom; ^8^Department of Disease Control and Prevention, School of Medicine and Health Sciences, Eden University, Lusaka, Zambia; ^9^Department of Paraclinical Studies, School of Veterinary Medicine, University of Zambia, Lusaka, Zambia; ^10^Africa Center of Excellence for Infectious Diseases of Humans and Animals, The University of Zambia, Lusaka, Zambia; ^11^Department of Arctic and Marine Biology, Faculty of Biosciences, Fisheries and Economics, UiT the Arctic University of Norway, Tromsø, Norway

**Keywords:** anti-bodies, human *Brucella*, risk factors, seroprvalence, Zambia

## Abstract

**Background:** Brucellosis is a neglected debilitating zoonosis widely recognized as an occupational health hazard. The seroprevalence of human anti-*Brucella* antibodies in high-risk populations, as well as their risk factors, have not been well-documented in Zambia. This study aimed at estimating the *Brucella* seroprevalence in herdsmen and abattoir workers and assess the associated risk factors.

**Methods:** A cross-sectional seroepidemiological study was carried out between May and December 2020 among abattoir workers and herdsmen in Namwala, Monze and Choma districts of Southern Province in Zambia. Seroprevalence was assessed by indirect enzyme-linked immunosorbent assay (i-ELISA) or competitive enzyme-linked immunosorbent assay (c-ELISA) while a questionnaire was administered to obtain epidemiological data.

**Results:** A total of 153 individuals were recruited in the study. The overall *Brucella* seroprevalence was 20.3% (95% CI: 14.6–27.5). Seropositivity among herdsmen and abattoir workers was 14.4% (95% CI: 9.2–21.8) and 46.4%, (95% CI: 28.8–65.0), respectively. Comparable seropositive results among districts showed Namwala with 26.9%, which was the highest, seconded by Monze 19.0%, and the least was Choma with 11.36%, seropositivity. The multivariate logistic regression model showed that occupation, age category, and district of residence were predictors of being seropositive to *Brucella* spp. antibodies. The odds of abattoir workers being seropositive to *Brucella* antibodies were 8.6 (95% CI: 2.6–28.2) higher than that of herdsmen being the reference group. The odds of age category 17–50 years being seropositive to *Brucella* antibodies were 7.0 (95% CI: 0.7–72.2) higher than being <16 years as the reference group. The odds of one having attained primary level of education being seropositive to *Brucella* were 1.3 (95% CI: 0.1–14.7) or secondary level of education were 6.2 (95% CI: 0.5–72.6) or tertiary level of education were 5.1 (95% CI: 0.2, 113.3) higher than that of no level of education as the reference group. Furthermore, the odds of a respondent being seropositive to *Brucella* antibodies were 4.5 (95% CI: 1.3–15.7) for Namwala and 4.9 (95% CI: 1.1–21.7) for Monze higher than that of Choma as the reference group.

**Conclusion:** Anti-*Brucella* antibodies are prevalent among herdsmen and abattoir workers in the study areas of Zambia (20.26%), a sign of exposure to *Brucella* pathogens. Type of profession, age and level of education seem to influence the exposure to Brucella pathogens. This zoonosis should be considered as one of the differential diagnosis in humans presenting intermittent fever, malaria-like signs and general pain in humans.

## Introduction

Human brucellosis is an infectious occupational disease, prevalent in Sub-Saharan African countries and typically caused by *B. abortus, B. melitensis, B. canis* and *B. suis* (1). The disease is listed as one of the seven neglected zoonotic diseases by the World Health Organization (WHO) (2). In humans, it usually originates from an animal reservoir (3). Brucellosis mainly affects high-risk occupational groups such as veterinarians, laboratory personnel, abattoir workers, slaughterhouse personnel, livestock keepers and farmers (4). These individuals get infected through inhalation of infectious aerosols, direct contact with infected animals/carcasses, or their products (raw milk, cheese and unpasteurized milk) (5). Brucellosis displays non-specific acute symptoms such as intermittent fever, backache, headaches, anorexia, weight loss, weakness and arthralgia (6). These symptoms are also seen in other diseases such as Malaria and Typhoid leading, therefore, to misdiagnosis and wrong therapy (7). Before the discovery of antibiotics, human brucellosis was described as “the disease rarely kills anybody, but it often makes a patient wish he were dead” (TIME magazine 1943). Human *Brucella* seroprevalence has been documented in different parts of the world among highly occupational groups which are comparatively mentioned as follows: China 15.5% (8); India 4.96% (9); Pakistan 18% (10); Malaysia 5.4% (11); Saudi Arabia 33.9% (12); Greece 3% (13); Egypt 31.3% (14); South Sudan 33.3% (15); Nigeria 24.1% (16); Cameroon 5.6% (17); Kenya 5.7% and 31.8% (18); Uganda 17% (19) and Tanzania 1.41% (20). In Zambia, there is a scarcity of data on human *Brucella* infections although seroprevalence has previously been estimated to be at about 5.03 % among livestock farmers in rural communities (21). Most health facilities in developing countries, including Zambia, rarely carry out routine brucellosis screening, therefore, the disease may be misdiagnosed and mistreated as other febrile diseases such as malaria (22) and underreported (23).

The seroprevalence and the associated risk factors of human infection in high-risk populations have not been well-understood and documented in the Southern Province of Zambia. Yet, a recent brucellosis study conducted in cattle in the same province found a herd seroprevalence of 28.5% (24). Considering that more than a third of Africa's population depend solely on livestock and livestock products for their livelihoods (25), the likelihood of human infection is therefore high. This is because most infected animals with brucellosis in Africa are not culled due to the economic consequences (26). This has led to the endemicity of human brucellosis in Africa (27) since livestock are the main source of infection to humans (22).

Although brucellosis is of great public health and economic concern, there has never been a brucellosis livestock mass vaccination campaign, making the current epidemiological situation in Zambia uncertain. Furthermore, laboratory diagnostic capacity is very weak mainly relying on rapid agglutination tests for diagnosis (28). These serological screening tests and results are insufficient in providing satisfactory evidence to attract any policies that would direct and reinforce control strategies both in human and livestock. Exploration of this health problem could give evidence-based data that would guide interventions since brucellosis requires multidisciplinary control approach. This study was therefore carried out to determine the *Brucella* seroprevalence among abattoir workers and herdsmen of Namwala, Monze and Choma districts as well as to find out factors associated with *Brucella* seropositivity.

## Materials and Methods

### Study Areas

**Figure 1:**
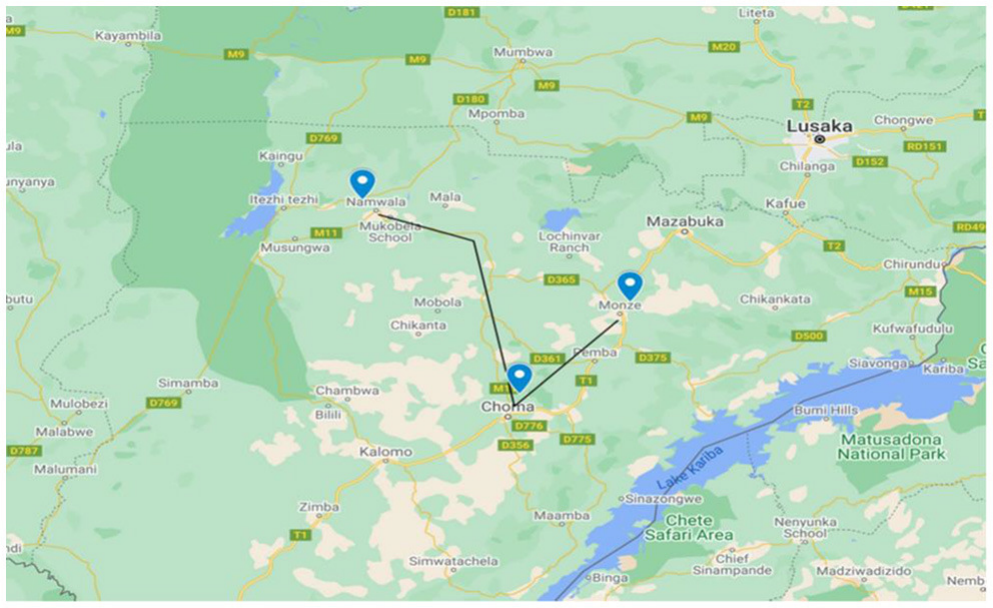
Map of the study areas (Choma, Monze and Namwala). Source: Zambia maps.

A cross-sectional study was carried out in Namwala, Choma and Monze districts of Southern province of Zambia (Figure 1). The province and study districts were purposively selected because they are the top cattle producers in Zambia (29). Furthermore, these areas are endemic for bovine brucellosis as documented by several serological surveys (24, 29, 30). Southern Province lies between latitudes 15°14′ S and 17°42′ S and longitudes 25°E and 28° S. It has a total land surface area of 85,283 Km^2^ with an estimated human population of 1,907,784 and a cattle population of 2,105,891 (31). In these districts, a pastoral or nomadic cattle-grazing system is practiced, where animals are grazed in the Kafue flats/floodplains in dry seasons and moved to the upper areas during the wet season (30).

### Study Population

The study population comprised of persons occupationally at risk of exposure to *Brucella* infections in Namwala, Choma and Monze districts of Southern province of Zambia. The individuals were grouped into two categories (herdsmen and abattoir workers), depending on their level of daily activities that could lead to direct contact with either suspected *Brucella* infected animals or infected animal carcass.

### Study Design and Sample Size Calculation

A sample size of 153 was calculated using the Ausvet EpiTools software (http://epitools.ausvet.com.au/) based on the following assumptions: Assumed prevalence estimation of 5.03% (21), desired absolute precision of +/−2% and confidence level of 95%. Sampling was stratified according to the study districts and weighted using the cattle population as proxy weighting value for the persons to be sampled (Table 1).

**Table 1:** Sample size of humans weighted against cattle population per district.

**District**	**Cattle population**	**Weighting index (human population)**	**Number of person to be sampled**
**Namwala**	145,704	102,866	50
	172,994	191,872	55
**Choma**	134,252	180,873	50
All areas	**452,950**	**475,611**	**155**

*The bold values indicate the proxy weights (Cattle versus human population) for individual participants to be sampled*.

### Sampling Technique and Sample Collection

A total of 153 individuals (125 herdsmen and 28 abattoir workers) were sampled from three purposively selected districts, namely Namwala, Monze, and Choma. Furthermore, these districts are subdivided into villages namely; Simaunbi, Mbabala, Mapanza, Katengwa, Maala, Batoka, Nakeempe, Kayuni, Muyoba, Siakasenke, Chitonga, Nakamboma, Nteme, Baambwe and Hakunkula. The herdsmen were selected based on their proximity to the abattoir, from locations adjacent to the abattoir to locations approximately 100 km from the abattoir. There were five active abattoirs in Namwala, five in Monze, and one in Choma. A list of all abattoir workers both in contact with the meat or slaughter of animals or both from an abattoir in each of the three districts, was obtained and used to randomly select individuals to screen. An informed consent was obtained from all study participants before blood collection. Four (4) ml of blood was collected via the median cubital vein by a clinical officer and stored in sterile plain tubes at +4°C. A semi-structured pre-tested questionnaire was then administered to collect information on the knowledge, attitudes, and practices of abattoir workers and herdsmen. The questionnaire was first developed in the English language and translated to the local language for better understanding of the questions by the participants.

### Laboratory Analysis

Serum samples from 153 participants were tested in parallel to detect anti-*Brucella* antibodies using the i-ELISA (Ingezim Brucella Compac 2.0, Madrid, Spain) and c-ELISA (SVANOVIR®Brucella-Ab Boehringer Ingelheim Svanova, Uppsala, Sweden) test kits according to manufacturer's instructions. On i-ELISA and c-ELISA tests, the result of the sample was compared with the mean cut-off value which was >40% mean optical density and >30% mean optical density of the four conjugate control wells, respectively. Any test sample giving an optical density equal to or below this value was recorded as being positive. In this study, parallel interpretation of results was used. Therefore, any sera testing positive either on i-ELISA or c-ELISA was regarded as positive.

### Data Analysis

The data obtained were coded and entered in the Microsoft Excel 2016®, exported, cleaned and analyzed using STATA version 14® (Stata Corp., College Station, TX, USA). Categorical data were expressed in percentage, and seroprevalence was calculated by dividing the number of positive sera samples by the total samples analyzed. Using the cut-off of mean optical density ≥30% and mean optical density ≥40%, for c-Elisa and i-ELISA, respectively, the independent effects of categorical risk factors on anti-*Brucella* spp. seropositivity were assessed using Fisher's exact test. Variables with a *p*-value ≤ 0.25 from univariate analysis were used as candidate variables in the logistic model. Multivariable logistic regression model was used to calculate odds ratio at 95% confidence interval to see the degree of association between *Brucella* seropositivity and the risk factors. The validity of the model to the observed data was assessed by computing the Pearson chi-square goodness-of-fit test.

### Ethical Consideration

Ethical clearance was obtained from Excellence in Research Ethics and Science (ERES) before commencing the study (Ref No. 2018-Dec-004). Permission to conduct the study was obtained from the Ministry of Health and the National Health Research Authority. The aim and brief background of the study were explained to the study participants in the local language, thereafter written informed consents were obtained for blood sample collection and questionnaire interview. Participants were assured of confidentiality, anonymity and were free to withdraw from the study whenever they chose to do so without incurring any consequence.

## Results

An overall *Brucella* seroprevalence of 20.3% (95% CI: 14.6–27.5) was estimated, based on parallel interpretation. The abattoir workers' category (*n* = 28) had a high *Brucella* seroprevalence (46.4%, *n* = 13, *p* < 0.001) as compared to the herdsmen (*n* = 125) category (14.4%, *n* = 18). Comparable seropositive results among districts showed Namwala with 26.9% (*n* = 18), which was the highest, seconded by Monze 19.0% (*n* = 8), and the least was Choma with 11.4% (*n* = 5) seropositive. *Brucella* seroprevalence of 5.2% (95% CI: 2.6–10.2) and 17.0% (95% CI: 11.8–23.9) were determined using c-ELISA and i-ELISA, respectively (Table 2). Namwala recorded high seroprevalences of 6.0 % (95% CI: 2.2–15.1) and 26.9% (95% CI: 17.5–38.9) for c-ELISA and i-ELISA; followed by Monze 4.8% (95% CI: 1.2–17.6) and 14.3% (95% CI: 6.5–28.7) for c-ELISA and i-ELISA, respectively. The least seroprevalence was recorded in Choma which was a duplicate value of 4.5% (95% CI: 1.1–16.8) for c-ELISA and i-ELISA, respectively. The difference between the groups (herdsmen and abattoir workers) was statistically significant (*p* < 0.001).

**Table 2:** Seroprevalence of human anti-*brucella* antibodies in Southern province by district among herdsmen and abattoir workers using c-ELISA or i-ELISA in parallel interpretation.

**District**	**c-ELISA (*****p*** **= 1.00)**		**i-ELISA (*****p*** **= 0.006)**		**Parallel Interpretation**
	**Positive**	**95% CI**	**Positive**	**95% CI**	
Namwala	4 (6.0%)	2.2–15.0	18 (26.9%)	17.5–38.9	18 (26.87%)
Monze	2 (4.8%)	1.2–17.6	7 (14.3 %)	6.5–28.7	8 (19.04%)
Choma	2 (4.5%)	1.1–16.8	2 (4.5%)	1.1–16.8	5 (11.36%)
Total	8 (5.2%)	2.6–10.2	27 (17.0%)	11.8–23.9	31 (20.26%)

Seropositive reactions among the abattoir workers and herdsmen varied according to the lifestyle and activities of the different categories of respondents. *Brucella* IgG seropositive respondents were 28 males and 3 females. The majority of the respondents (54.3%; *n* = 83), reported that they had never heard about brucellosis. Less than half (45.8 %; *n* = 70) of the participants were knowledgeable about the disease (Table 3). There was no association between *Brucella* seropositivity and some factors such as gender, knowledge and type of production among herdsmen and abattoir workers (Table 3). In contrast, there was a statistically significant association between *Brucella* seropositivity for herdsmen and type of breeding method (natural/both) (*P* = 0.02) as well as abattoir workers who, during their line of duty, had blood splashed around their mouth (*P* = 0.01).

**Table 3:** Univariate analysis for predicators of brucellosis among herdsmen and abattoir workers.

**Variable**	**Category**	**Total**	**Positive**		**95% CI**	*P*-value^*^
		***N* (153)**	***N***	**%**		
Occupation	Abattoir worker	28	13	46.43	28.79–65.00	0.00
	Herdsmen	125	18	14.4	9.21–21.80	
Gender	Male	144	28	19.44	13.72–26.82	
	Female	9	3	33.33	10.20–68.75	0.315
Age categories	0–16	4	2	50.00	9.27–90.73	
	17–50	118	28	23.73	16.84–32.34	
	>50	31	1	3.23	0.43–20.42	0.006
Level of education	No FE	7	1	14.29	1.64–62.56	
	Primary	91	12	13.19	7.59–21.94	
	Secondary	49	16	32.65	20.88–47.11	0.028
	Tertiary	6	2	33.33	7.13–76.52	
Knowledge of Brucellosis	No	83	14	16.87	10.18–26.65	
	Yes	70	17	24.29	15.55–35.84	0.255
Breeding method	*N* (125)					
	Natural	111	14	12.50	7.49–20.13	
	Both	9	4	28.57	10.61–57.41	0.02
Type of production	Communal	100	16	15.84	9.87–24.45	0.316
	Individual	25	2	8	1.92–27.82	
Blood around the mouth	*N* (28)					
	No	12	3	18.18	4.00–54.52	No
	Yes	16	11	68.75	41.18–87.36 0.01	Yes

* *Fishers exact test; CI, confidence interval*.

### Logistic Regression Model and Validation

**Figure 2:**
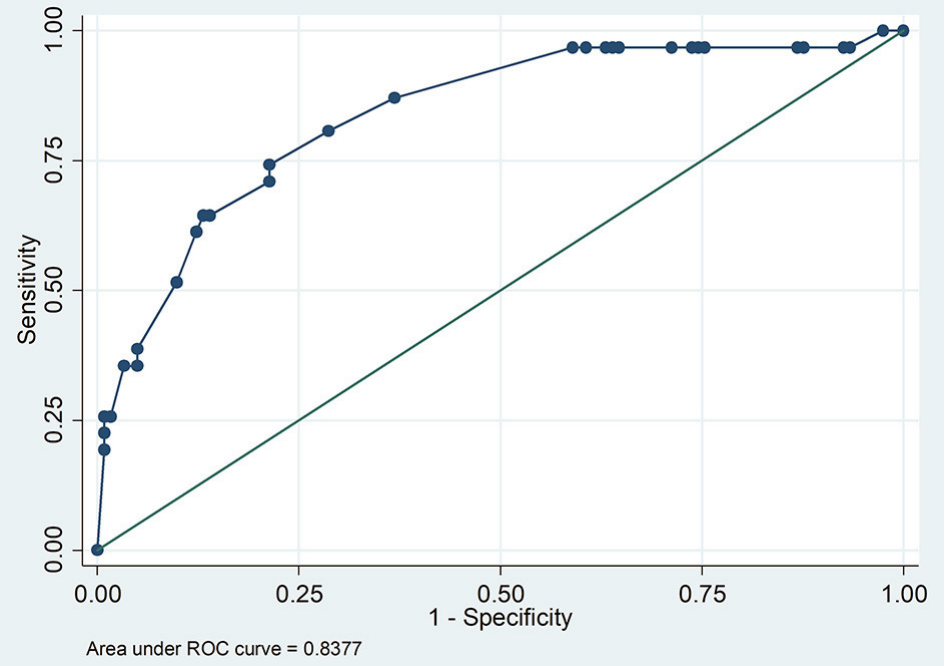
ROC curve demonstrating predictability of the model.

**Figure 3:**
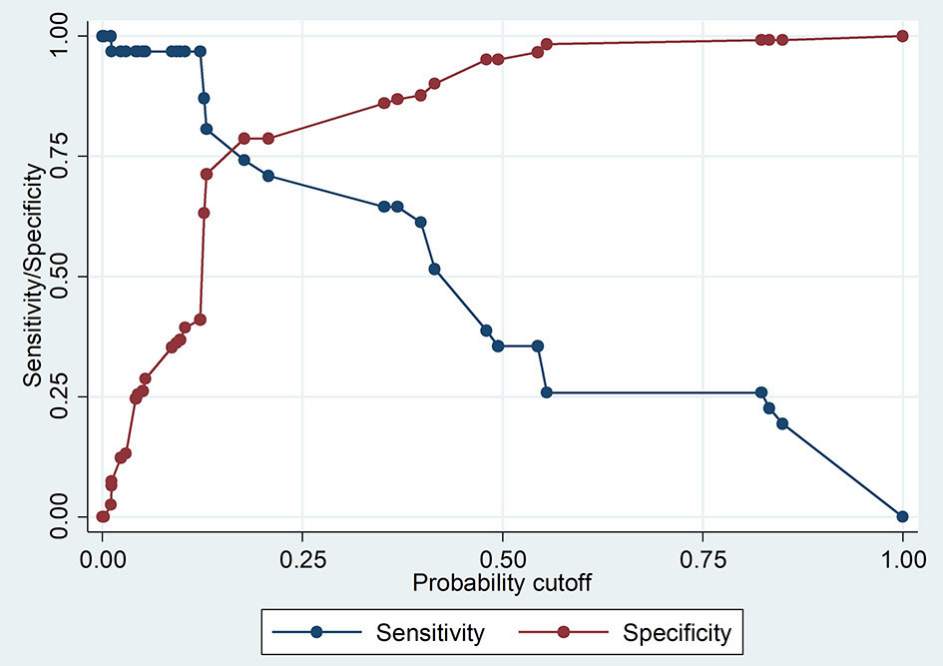
Graphs demonstrating probability cutoff vs. sensitivity and specificity.

The Pearson chi-square goodness-of- fit test (*p* = 0.1908) showed that the model fitted the data thus increasing its reliability in predicting. The Receiver-operating Characteristic curve ROC analysis demonstrated that the model was good in prediction (the area under the curve was 0.8377) (Figure 2). The model had rather high sensitivity and low specificity in classifying individuals as seropositive or seronegative (Figure 3).

The multivariate logistic regression model showed that occupation, age category, and district of residence were predictors of being seropositive to *Brucella* spp (Table 4). The odds of abattoir worker being seropositive to *Brucella* antibodies were 8.6 (95% CI: 2.6–28.2) higher than that of herdsmen being the reference group. The odds of age category 17–50 years being seropositive to *Brucella* antibodies were 7.0 (95% CI: 0.7–72.2) higher than being <16 years as the reference group. The odds of one having attained primary level of education being seropositive to *Brucella* were 1.3 (95% CI: 0.1–14.7) or secondary level of education were 6.2 (95% CI: 0.5–72.6) or tertiary level of education were 5.1 (95% CI: 0.2, 113.3) higher than that of no level of education as the reference group. Furthermore, the odds of a respondent being seropositive to *Brucella* antibodies were 4.5 (95% CI: 1.315.7) for Namwala and 4.9 (95% CI: 1.1–21.7) Monze higher than that of Choma as the reference group.

**Table 4:** Multivariable logistic regression analysis for brucellosis risk factors in humans.

**Variable**	**Level**	**Pos./Tested**	**Seropositivity (%)**	**Odd ratio**	**95% CL**	***P*-value**
Occupation	Herdsman	18/125	14.4	Ref		
	Abattoir worker	13/28	46.43	8.6	2.6–28.2	0.00
Age Category	<16 years	2/4	50.00	Ref		
	17–50 years	28/118	23.73	7.0	0.7–72.2	0.10
	>50 years	1/31	3.23	0.1	0.0–0.7	0.02
	No-education	1/7	14.29	Ref		
Level of education	Primary	12/91	13.19	1.3	0.1–14.7	0.84
	Secondary	16/49	32.65	6.2	0.5–72.6	0.15
	Tertiary	2/6	33.33	5.1	0.2–113.3	0.31
District	Choma	5/44	11.36	Ref		
	Namwala	18/67	26.87	4.5	1.3–15.7	0.02
	Monze	8/42	19.04	4.9	1.1–21.7	0.04

*Pos, Positivity; CI, Confidence interval; Ref, Reference*.

## Discussion

### Seroprevalence of Human *Brucella* Antibodies

The estimated seroprevalence of *Brucella* antibodies was 20.3% (14.4% in herdsmen and 46.4% in abattoir workers). This is higher than that reported in a previous studies undertaken between August 2004 and July 2005 in the Southern province of Zambia, which found a 5.03% seroprevalence in humans (21). However, the cited study did not include abattoir workers, but rather focused only on livestock farmers. Abattoir workers were at an increased risk of exposure to *Brucella* because farmers in the study area tend to cull animals that are old or have reproductive challenges by slaughtering them. Furthermore, during the slaughtering process, abattoir workers are constantly exposed to aerosols and animal parts i.e., blood, tissues, fluids, with inadequate or poor use of personal protective equipment. Further, these workers were at a high risk of injury (knife-cuts) as compared to herdsmen, which increases the risk of exposure to the *Brucella* pathogen (20). A *Brucella* cattle herd seroprevalence of 28.5% has been previously been estimated in this study area (24). Comparable seropositive results among the study districts showed that Namwala had the highest (26.8%), followed by Monze (19.4%), while Choma had the lowest (11.4%). The high seropositivity in Namwala district can be attributed to its close proximity to the Kafue flood plains where most of the livestock come into close contact with wild animals. Migration of livestock from the sampled districts to the Kafue plains in search of pasture during the dry season is regular and usual phenomenon due to drastic change in the weather conditions thus increasing co-mingling of animals and transmission of infectious diseases such as brucellosis. Herdsmen tend to drink raw milk, hence the increased likelihood of exposure (5). The difference observed may also be due to the use of different diagnostic test and also the difference in the environmental condition of the region.

Our findings are higher than the 1.41% reported among butcher men, abattoir workers and herdsmen in Tanzania (20) and the 5.6 and 4.7% among abattoir worker in Cameroon (17) and Ethiopia (32), respectively. Handling of fetus and uterine contents was associated with increased risk of human brucellosis in Cameroon whereas in Ethiopia this was attributed to low levels of disease awareness and one working in the abattoir.

Similarly, our findings are higher than the reported seroprevalences among slaughterhouse workers (9.6%) in Ghana (33) and livestock farmers (4.4%) in Uganda (34) but lower than 33.3% observed among cattle herders in South Sudan (15). In contrast, our findings are comparable to 18.5% by (35), higher than the 33.5% reported among abattoir workers in Nigeria (36) and higher than the 7.9% among butcher and slaughterhouse workers in Iran (37).

One of the limitations of this study was that formal random sampling was not achieved as inclusion in the study was voluntary. Further, the participants in this study were not clinically examined for evidence of brucellosis. Therefore, the estimated seroprevalence does not imply persons were suffering from brucellosis, but that the persons had anti-*Brucella* antibodies, thus likely to be infected. Despite this, the results give a good indication of brucellosis situation in the sampled districts. In endemic areas, as prevailing in the study areas, cases of sub-clinical and self-limiting episodes of brucellosis are likely to show anti-*Brucella* spp. antibodies thus reducing assay specificity (38). However, the seroprevalence found in the current study parallels with bovine herd *Brucella* seroprevalence that has been reported to be 28.5% (24) and 20.7% by (29). It is most probable that the Brucella infected animals in our study area were likely to act as reservoirs for human brucellosis (39). Thus, high bovine *Brucella* seroprevalence in the region could explain the possible reason for the high seroprevalence in humans.

### Risk Factors Associated With *Brucella* Seropositivity

In this study, occupation, age category and district of residence were identified as risk factors for human brucellosis. Our current study revealed that seroprevalence was highest among abattoir workers 46.43% (*n* = 13). The odds of disease were 8.6 times higher in abattoir workers than in herdsmen, implying the former were more at risk.

The 17–50 years' age category (considered to be actively working with livestock) was the most commonly affected with *Brucella* in the study area although this was not statistically significant. Furthermore, a respondent who was in the 17–50 years' age category was 7 times more likely to be infected with brucellosis than those who were <16 years old (school going children). These did most of the physical work requiring direct contact with the livestock and livestock products. Similarly (17), reported an association between age and an increased seroprevalence. Similar findings have been reported in Pakistan where age was statistically associated to *Brucella* seropositivity (40).

Namwala district had 4-fold higher risk of brucellosis than Choma. This might be due to the fact that Namwala has the highest cattle density in the Country and animals are communally grazed on the Kafue plains (30). Furthermore, Mfune et al. (24) established that the odds of testing positive for *Brucella* in animals were high in Namwala district (OR = 8.55, CI: 2.66–27.44) compared to those from other districts.

A noteworthy association is the type of husbandry practices (Communal/individual) coupled with contact with the Kafue lechwe which have been documented to harbor brucellosis on the flood plains of the Kafue River in this ecosystem (30, 41). Assenga et al. (39) demonstrated the presence of anti-Brucella antibodies in humans, livestock, and wildlife in the Katavi- Rukwa ecosystem in Tanzania. Transmission of the infection between wildlife, livestock and humans is likely to continue due to increasing human activities in the human wildlife interface.

However, the spread out large odds ratios, with wide confidence intervals obtained in this study should be cautiously interpreted, given that the distribution of the individuals within the two occupational categories of the risk factors was not even.

Although this study implicated livestock breeding method (natural breeding) (*p* = 0.02) as a probable risk factor associated to *Brucella* seropositivity, Nguna et al. (34) reported that livestock breeding method was not an important risk factor among the herdsmen. Communal grazing coupled with natural breeding is widely practiced hence the risk of transmission of disease to susceptible animals. Similar studies were done by Kubuafor et al. (42) where a significant association between antibodies against *Brucella* and a history of abortions and retained placenta was observed.

History of blood splashes around the mouth was observed to be associated with seropositivity among abattoir workers (*p* = 0.01). Similar findings have been documented in Southwest Nigeria (43), Tanzania (44) and Egypt (45). Noteworthy difference is that breeding method and blood around the mouth were potential risk factors and statistically significant at univariate analysis but were dropped in the final multivariable logistic regression model. This was done to increase the predictability of the final model.

The odds of disease in participants older than 50 years was <1, implying old-age was a protective factor. In spite of the high seroprevalence of *Brucella* infection in humans, it was not considered for routine laboratory diagnosis in cases of acute febrile illness.

## Conclusion

Anti-*Brucella* antibodies in herdsmen and abattoir workers was prevalent in Southern province of Zambia (20.3%), an indication of exposure to Brucella pathogens. The seroprevalence was higher than that observed in similar studies in Zambia. The majority of the respondents, 54.25% (*n* = 83) reported having not heard of brucellosis. Less than half 45.75 of % (*n* = 70), of the participants were knowledgeable of the disease, thus it can be concluded that community knowledge about the risk factors of human brucellosis was poor. The important predicators of *Brucella* seropositivity were occupation, age category and district. This zoonosis should always be one of the differential diagnosis in humans when intermittent fever, malaria like signs and general pain are observed in humans.

## Study Limitations

The Covid-19 pandemic, outbreak of Foot and Mouth Disease in the study areas and the instability in some parts of the country which was attributed to terrorist gas-attacks during the sampling period made it difficult to collect human and cattle blood samples. Most individuals were not willing to participate in the study, alleging that it was against their religious and cultural beliefs for blood to be drawn from them. Thirdly, since our study did not present the bacteriological evidence or molecular-based tests, the seropositivity results might be caused by previous exposure to infection or cross-reactivity. However, c-ELISA used in this study is known to have high specificity.

## Data Availability Statement

The original contributions presented in the study are included in the article/supplementary material, further inquiries can be directed to the corresponding author/s.

## Ethics Statement

The studies involving human participants were reviewed and approved by Excellence in Research Ethics and Science (ERES). Written informed consent to participate in this study was provided by the participants' legal guardian/next of kin.

## Author Contributions

MM and RM: conceptualization and validation. MM, JBM, and RM: methodology. MM, JBM, MS, and FB: software. MM, JBM, and JG: formal analysis. MM, RM, and CC: investigation. RM: resources and project administration. MM, JBM, and JK: data curation. MM: writing—original draft preparation and visualization. RM, JK, AM, JM, BH, JG, and JBM: writing—review and editing. JBM, RM, and JG: supervision. JBM: funding acquisition. All authors contributed to the article and approved the submitted version.

## Funding

This work was funded by the African Centre for Infectious Diseases in Humans and Animals UNZA (ACEIDHA-UNZA).

## Conflict of Interest

The authors declare that the research was conducted in the absence of any commercial or financial relationships that could be construed as a potential conflict of interest.

## Publisher's Note

All claims expressed in this article are solely those of the authors and do not necessarily represent those of their affiliated organizations, or those of the publisher, the editors and the reviewers. Any product that may be evaluated in this article, or claim that may be made by its manufacturer, is not guaranteed or endorsed by the publisher.
